# Chronic Urticaria and Its Impact on the Quality of Life of Nepalese Patients

**DOI:** 10.1155/2020/6694191

**Published:** 2020-11-28

**Authors:** Sushil Paudel, Niraj Parajuli, Rabindra Prasad Sharma, Sudip Dahal, Sudarshan Paudel

**Affiliations:** ^1^Department of Dermatology, Civil Service Hospital, Kathmandu 44600, Nepal; ^2^Department of Dermatology, National Academy of Medical Sciences, Kathmandu 44600, Nepal; ^3^Department of Statistics, Civil Service Hospital, Kathmandu 44600, Nepal; ^4^School of Public Health, Patan Academy of Health Sciences, Lalitpur 44600, Nepal

## Abstract

Chronic urticaria (CU) is a skin condition characterized by sudden and recurrent episodes of wheals, angioedema, or both and commonly associated with itching for a duration of more than six weeks. The available data indicate that urticaria markedly affects both objective functioning and subjective well-being of patients. A review of patients' records with chronic urticaria attending Civil Service Hospital from January 2018 to December 2019 was done. A detailed demographic data of all patients with chronic urticaria was also retrieved. Dermatology Life Quality Index questionnaire (DLQI) Nepalese version was used for the assessment of the impact of disease on life quality. Mann–Whitney *U*-test was applied to compare means, and principle component analysis for factor analysis was used. A total of 149 patients were included, with a male-to-female ratio of 1 : 1.9. The mean age of the study population was 32.86 ± 12.837 years. The mean DLQI score was 8.30 ± 6.73 with men having a significantly greater score than women (*p* < 0.02). DLQI scores negatively correlated with age (*p* < 0.01). There was a high internal consistency among items (Cronbach's alpha 0.89), and all items had satisfactory correlation with each other as well. Principle component extraction revealed that there were two underlying factors in the DLQI questionnaire on measuring quality of life in chronic urticaria. Males had a greater impairment in quality of life than females due to chronic urticaria. Most severe impairment was seen in symptoms/feelings subdomain. It also revealed that there were two different underlying factors in DLQI questionnaire.

## 1. Background

Chronic urticaria (CU) is a skin condition characterized by pruritus with sudden and recurrent episodes of wheals, angioedema, or both for more than six weeks [1]. The worldwide prevalence of chronic urticaria is estimated to be at 1% and that of acute urticaria is in a range of 10–20% [2]. A single study conducted in rural Nepal estimated the prevalence of urticaria to be at 2.4% [3]. The available data till now have indicated that urticaria markedly affects both objective functioning and subjective well-being of patients [4–6]. Health status scores in CU patients were comparable with that of patients with coronary artery disease [7], but lower than those with respiratory allergy, which showed a significant impact on the patient's life quality [8].

The Dermatology Life Quality Index (DLQI) was the first dermatology specific instrument developed to assess the impact of skin diseases in an individual's life based on the experiences of the previous seven days [9]. It is a validated tool to measure the quality of life and has been widely used in more than 30 different skin conditions and translated in more than 50 languages including Nepali [10, 11]. It has also been used in evaluating the treatment efficacy or intervention in patients with chronic urticaria [12–17].

However, there is a paucity of data regarding its impact in the lives of Nepalese population. This study endeavored to evaluate the impairment of different aspects of life in patients with chronic urticaria.

## 2. Materials and Methods

This was a retrospective review of records of patients visiting outpatient department of dermatology in Civil Service Hospital, Kathmandu, from January 2018 to December 2019 with chronic urticaria. All the demographic details and details from the DLQI-Nepali version filled up by consenting patients as part of the initial assessment for the treatment of CU were reviewed and included in this study. All patients, more than 16 years of age, with the diagnosis of chronic spontaneous urticaria were included in this study. Exclusion criteria included patient less than 16 years old, incomplete DLQI forms, patients presenting with only angioedema or only chronic inducible urticaria, and patients with other chronic diseases that could impact the quality of life. The DLQI-Nepali questionnaire was translated by Dr. Sudha Agrawal and was downloaded from https://www.cardiff.ac.uk/medicine/resources/quality-of-life-questionnaires/dermatology-life-quality-index for the purpose of this study.

There are 10 questions or items in the DLQI questionnaire, which are further subdivided into six subdomains of life. Items 1 and 2 pertain to symptoms and feelings; items 3 and 4, to daily activities; items 5 and 6, to leisure; item 7, to work and school; items 8 and 9, to personal relationships, and item 10 pertains to treatment. There are four choices to choose from in response to each of the items: “not at all,” “a little,” “a lot,” and “very much” which are scored as 0, 1, 2, and 3, respectively. Some of the items contain a fifth option, “not relevant” which is scored as 0. The maximum score of DLQI reaches up to 30. A higher score indicates greater impairment of quality of life. For ease of interpretation, the scores are banded in six categories each representing different severities of the disease. In summary, a total score of 0-1 signifies no impairment; 2–6, mild impairment; 6–10, moderate impairment; 11–20, severe impairment; and 21–30, very severe impairment in QoL.

Based on the distribution of the variables mean ± standard error (SE) and median values were calculated. Student's *t*-test or Mann–Whitney *U*-test were used to find significant differences in the means, and correlations between continuous variables were tested. Normality of the distribution of variables was tested by Shapiro–Wilk test (*p* < 0.05 rejecting the hypothesis of normality). The internal consistency (reliability) of the Nepali version of DLQI in chronic urticaria was tested using Cronbach's alpha, and the value of alpha greater than .70 was considered satisfactory. Exploratory factor analysis was performed by the principle component analysis method to find the underlying factor(s) structure in DLQI. In all tests, *α* was set at 5%. Statistical tests were performed using the Statistical Package for the Social Sciences (SPSS) version 25.

## 3. Results

In total, 149 patients with CU fulfilled the inclusion criteria and were included in the final evaluation. Females outnumbered males, with a male-to-female ratio of 1 : 1.9. The mean age (±SE) of the study population was 32.86 ± 1.05 years. Age of the females (34.66 ±  1.29) was significantly higher than that of the males (29.39 ± 1.71), (*p*=0.017). The mean duration (±SE) of disease in males was 15.37 ± 3.07 months and that in females was 13.28 ± 1.94 months. However, the difference was not statistically significant (*p*=0.91). The median duration of the disease, in the study population, was 6 months, and the range was from 1.5 months to 120 months.

### 3.1. Dermatology Life Quality Index Scoring

The mean DLQI score was 8.30 ± 0.55, which indicated that the impact of chronic urticaria was moderate in the study population. According to the sex, mean DLQI score was 10.51 ± 1.1 (range: 1–28) in males and 7.15 ± 0.58 (range: 1–21) in females (*p*=0.026). The mean score was significantly higher in males than in females for questions/items 6 to 10 (Table 1).

Among the six subdomains of life quality measured by the questionnaire, four of them showed that males were more severely affected in comparison with females by CU (*p* < 0.05) (Table 2).

**Figure 1:**
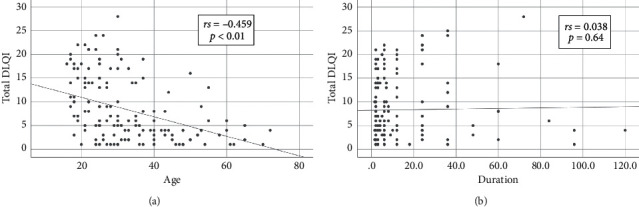
Bivariate correlation between the DLQI score and (a) the age of the patient showing a significant negative correlation between the two and (b) the duration of the disease showing no correlation between the two.

On correlating DLQI score with the age and duration of disease, there was a decreasing DLQI score with an increase in age (*r*_*s*_ = −0.459; *p* < 0.01). Younger patients had significantly more impaired QoL as compared to adults (Figure 1(a)). But, no correlation was found between the duration of disease and the DLQI score (*r*_*s*_ = 0.038; *p*=0.64) (Figure 1(b)).

### 3.2. Test for Internal Consistency

The internal consistency among the questions (items) was found to be high. Cronbach's alpha was 0.88, which became 0.89 after standardization, and it did not change with the deletion of any of the items which is higher than the traditionally accepted level of 0.70. The interitem correlation matrix revealed that the correlation coefficients (*r*) ranged from 0.097 to 0.730 (Table 3). All items had a satisfactory correlation with each other. Thus, all the items were included for the further factor analysis.

### 3.3. Factor Analysis

Principle component analysis method was used for factor analysis. Bartlett's test of sphericity showed *χ*^2^ = 781.19 (*p* < 0.001); the Kaiser–Meyer–Olkin measure of sampling adequacy had a value of 0.89, which indicated that this dataset could be analyzed for factor analysis. Principle component extraction method revealed that there are two factors with the initial eigenvalues of more than 1 and a cumulative contribution of 65.91% (Table 4). These results mean that the DLQI has two factors (underlying dimensions) while measuring chronic urticaria. The cutoff value for the item loading (*α* coefficient) was set at 0.4. The Varimax rotation with Kaiser normalization resulted component matrices of the items as shown in Table 4. Items 1 to 3 had large loading on factor (component) 1, and items 4 to 10 loaded on factor 2. Items 4 and 5 had higher load on factor 2, though their load on factor 1 was more than 0.5 and suggested to have a shared loading on both factors.

## 4. Discussion

CU was more common in females (male : female = 1 : 1.9), which is similar to the findings from other similar studies conducted in India [16], China [17], Italy [18], Germany [19], and Korea [20]. The mean age of patients suffering from CU was 32.86 ± 12.83 years, similar to that from China.

However, the mean age was lower in Japan [15], where it was 45.2 ± 11.3 years, and in Germany [19], where it was 42.17 ± 9.24 years. The mean age of males was significantly lower than females in this study, which has not been reported by other studies.

In our study, the mean total DLQI score was 8.30, indicating a moderate impairment in the QoL. This score is close to studies done by Basra et al. [11], where he analyzed studies conducted in 12 different countries with a mean DLQI of 9.80 with a range of 7.16–13.40. Similar scores were also noted in a study conducted in China (mean DLQI score = 9.93) [17]. In contrast, the mean DLQI score was lower in India (mean = 6.63) [16] and Japan (mean score = 4.8) [15]. Males were affected significantly more by CU than females (*p* < 0.05) in our study, which was different from other studies [16, 17, 19, 21]. However, Poon et al. [22] found that there was no difference in total DLQI score between the genders.

The two biggest impacts were in the subdomains related to symptoms and feelings (57.66%) and work/school (30.33%). Personal relationship (9.5%) and treatment (16%) subdomains were the two least affected. These findings were similar to findings from study in China [17].

Males were significantly more affected in four out of six subdomains, namely, leisure (*p*=0.005), work/school (*p*=0.001), personal relations (*p*=0.004), and treatment (*p*=0.033) in our study. Similar results were also derived in a study by Poon et al., where leisure was more impaired in men [22]. However, a study from China [17] showed that school/work subdomain was significantly more impaired in females.

Our study found that the QoL was more impaired in younger age. This finding was consistent with some studies [17, 19], but opposite to others [22].

DLQI being a generic skin HRQoL measurement can take various dimensionalities, and its reliability could differ from the disease under study and the population under study [11].

The reliability for the Nepalese version of DLQI was found to be high. When examined for dimensionality, it was found to have two dimensions while measuring the CU in Nepalese population. Items 1 to 3 had a large load on factor 1 and items 6 to 10 had loads on factor 2. Items 4 and 5 had a shared loading on both factors. Item 4, named clothing in the original version of DLQI, more aptly fits with the other items of factor 1. When internal consistency was tested with item 4 in factor 1, Cronbach's alpha was satisfactory for both factor items (Table 5). These findings suggest that the impact of CU as measured by DLQI in Nepalese population has at least two dimensions. Factor 1 comprises the items representing symptoms and embarrassing situations that itching and wheals can produce and be labelled as “itching/embarrassing,” and factor 2 comprises the items related to different social and personal functions, so it can be labelled as “functioning”.

Similar two-dimensionality of DLQI in CU was found in Chinese version [17] also, but due to the low correlation coefficient between the items, they eliminated item 1 from factorization and found items 2 to 6 loaded on factor 1 and items 7 to 10 on factor 2. However, the study by Lennox et al. [14] had found that the DLQI is a one-dimensional measure of QoL.

## 5. Conclusion

The males were more severely affected by CU in comparison with females in our study. The impact of CU in Nepalese population as measured by DLQI had two dimensions of affliction. These findings need to be reevaluated in a larger population. There is a need for further studies in other skin conditions apart from CU and a larger set of populations for a proper validation of the Nepali version of DLQI in our population.

## Figures and Tables

**Table 1:** The mean DLQI scores for each item (questions) and their comparison with the gender Mann–Whitney *U*-test (*p* value).

DLQI questions	Mean score			*p* value
Shortened names	Male	Female	All	
(1) Symptoms	2.14	2.33	2.26	0.187
(2) Feeling	1.31	1.15	1.21	0.478
(3) Daily activities	1.12	0.94	1.00	0.347
(4) Choice of clothing	0.82	0.53	0.63	0.083
(5) Social/leisure	0.80	0.52	0.62	0.160
(6) Sports	0.94	0.40	0.58	**0.001**
(7) Work/study	1.69	0.51	0.91	**0.001**
(8) Relationships	0.65	0.23	0.38	**0.003**
(9) Sexual difficulties	0.35	0.12	0.20	**0.019**
(10) Treatment	0.67	0.40	0.49	**0.033**
Total	10.51	7.15	8.30	**0.026**

The mean scores of items 6 to 10 were significantly higher in males compared to females (p < 0.05), indicating males were more severely affected by CU in those areas.

**Table 2:** The mean score ± standard error by the subdomains of the DLQI in patients with chronic urticaria.

Subdomains (full marks)	Male (%)	Female (%)	All patients (%)	*p* value
Symptoms and feelings (6)	3.45 ± 0.24 (57.5)	3.47 ± 0.15 (57.83)	3.46 ± 0.13 (57.66)	0.859
Daily activities (6)	1.94 ± 0.27 (32.33)	1.64 ± 0.17 (27.33)	1.63 ± 0.13 (27.17)	0.204
Leisure (6)	1.74 ± 0.25 (29.00)	0.91 ± 0.13 (15.16)	1.20 ± 0.12 (20.00)	**0.005**
Work and school (3)	1.68 ± 0.19 (56.00)	0.51 ± 0.10 (17.00)	0.91 ± 0.10 (30.33)	**<0.001**
Personal relationships (6)	1.00 ± 0.22 (16.66)	0.35 ± 0.10 (5.83)	0.57 ± 0.10 (9.50)	**0.004**
Treatment (3)	0.66 ± 0.13 (22.00)	0.39 ± 0.08 (13.00)	0.48 ± 0.07 (16.00)	**0.033**
Total score (30)	10.51 ± 1.10 (35.03)	7.15 ± 0.58 (23.83)	8.3 ± 0.55 (27.67)	**0.026**

Mann–Whitney *U*-test (*p* value). Out of six sub-domains of the DLQI questionnaire, four, namely. leisure, work and school, personal relationships and treatment were significantly more impaired in males compared to females.

**Table 3:** Interitem correlation matrix of the DLQI items.

	Q2	Q3	Q4	Q5	Q6	Q7	Q8	Q9	Q10
Q1	0.466	0.437	0.215	0.336	0.168	0.127	0.097	0.133	0.158
Q2		0.639	0.548	0.562	0.516	0.424	0.390	0.338	0.445
Q3			0.581	0.514	0.480	0.511	0.303	0.292	0.377
Q4				0.482	0.623	0.550	0.417	0.439	0.518
Q5					0.549	0.470	0.524	0.514	0.573
Q6						0.599	0.506	0.455	0.573
Q7							0.559	0.435	0.551
Q8								0.730	0.701
Q9									0.623

Q: question/item.

**Table 4:** Principal component analysis with Varimax rotation: loading of the items on the two factors.

Items	Factor 1	Factor 2
Q1		0.780
Q2		0.767
Q3		0.792
Q4	0.566	0.517
Q5	0.595	0.501
Q6	0.674	
Q7	0.687	
Q8	0.874	
Q9	0.816	
Q10	0.827	
% of variance	39.444	26.474
Cumulative (%)	39.444	65.918

**Table 5:** Internal consistency for 2 factors of Nepali version.

Factor	Items	Cronbach's alpha
1	1, 2, 3, 4	0.79
2	5, 6, 7, 8, 9, 10	0.86

## Data Availability

The data used to support the findings of this study are available from the corresponding author upon request.
